# Microglia Impairs Proliferation and Induces Senescence In-Vitro in NGF Releasing Cells Used in Encapsulated Cell Biodelivery for Alzheimer’s Disease Therapy

**DOI:** 10.3390/ijms23169011

**Published:** 2022-08-12

**Authors:** Sumonto Mitra, Ruchi Gera, Julia Sundheimer, Marine Lemee, Lars U. Wahlberg, Bengt Linderoth, Maria Eriksdotter, Homira Behbahani

**Affiliations:** 1 Division of Clinical Geriatrics, Center for Alzheimer Research, Department of NVS, Karolinska Institutet, 141 52 Huddinge, Sweden; 2 Gloriana Therapeutics, Inc., Providence, RI 02885, USA; 3 Department of Clinical Neuroscience, Karolinska Institutet, 171 77 Stockholm, Sweden; 4 Theme Inflammation and Aging, Karolinska University Hospital, 141 86 Huddinge, Sweden

**Keywords:** Alzheimer’s disease (AD), amyloid beta (Aβ), drug delivery, encapsulated cell biodelivery (ECB), inflammation, microglia, nerve growth factor (NGF), senescence, therapy

## Abstract

There is no cure yet available for Alzheimer’s disease (AD). We recently optimized encapsulated cell biodelivery (ECB) devices releasing human mature nerve growth factor (hmNGF), termed ECB-NGF, to the basal forebrain of AD patients. The ECB-NGF delivery resulted in increased CSF cholinergic markers, improved glucose metabolism, and positive effects on cognition in AD patients. However, some ECB-NGF implants showed altered hmNGF release post-explantation. To optimize the ECB-NGF platform for future therapeutic purposes, we initiated in-vitro optimization studies by exposing ECB-NGF devices to physiological factors present within the AD brain. We report here that microglia cells can impair hmNGF release from ECB-NGF devices in-vitro, which can be reversed by transferring the devices to fresh culture medium. Further, we exposed the hmNGF secreting human ARPE-19 cell line (NGC0211) to microglia (HMC3) conditioned medium (MCM; untreated or treated with IL-1β/IFNγ/Aβ_40_/Aβ_42_), and evaluated biochemical stress markers (ROS, GSH, ΔΨ_m_, and Alamar Blue assay), cell death indicators (Annexin-V/PI), cell proliferation (CFSE retention and Ki67) and senescence markers (SA-β-gal) in NGC0211 cells. MCMs from activated microglia reduced cell proliferation and induced cell senescence in NGC0211 cells, which otherwise resist biochemical alterations and cell death. These data indicate a critical but reversible impact of activated microglia on NGC0211 cells.

## 1. Introduction

Presently, there are two classes of drugs in clinical use worldwide to treat Alzheimer’s disease (AD), namely cholinesterase enzyme inhibitors (ChEIs; naturally derived, synthetic and hybrid analogues) and antagonists to N-methyl D-aspartate (NMDA) receptors. Recently, the anti-amyloid beta (Aβ) antibody, Aducanumab, was approved for AD therapy by the United States of America Food and Drug administration (U.S. FDA) but subsequently failed to secure approval from the European Medicines Agency (EMA). Since the discovery of AD, numerous studies have aimed to find a cure for AD to reduce symptoms of the disease, utilizing different clinical and pathological perspectives.

Various reports link AD progression with the declining activity of cholinergic neurons in the basal forebrain, partly due to the inefficiency of nerve growth factor (NGF) maturation [1]. Neurotrophic factors, such as NGF, have been found in different areas of the nervous system as well as in cerebrospinal fluid (CSF) [2]. NGF regulates differentiation, growth, survival, and plasticity of basal forebrain cholinergic neurons (BFCNs) [3,4]. Due to its multifarious biological action, NGF has been utilized as a therapeutic factor in clinical trials of varied diseases, including AD [5]. NGF as a disease-modifying therapy in human has had challenges regarding delivery limitations, due to its inability to cross the blood-brain barrier and systemic side effects. Therefore, the delivery of NGF to the brain for therapeutic purposes has been explored by different methodological approaches in AD [6,7]. Important examples include the intranasal administration of an engineered form of human NGF-61 (hNGF-61) [8], direct injection into the cerebral lateral ventricle of the AD brain [9], gene therapy approaches [7,10,11,12,13], and the stem-cell-based delivery [14,15].

Previously, our group utilized NGF in clinical trials with the intention to improve cholinergic neurons health and function in the basal forebrain of AD patients. In 2012, we presented a new approach—encapsulated cell biodelivery (ECB) technology, as an alternative to deliver human mature (hm) NGF, aiming to halt the degeneration of cholinergic neurons in the basal forebrain and improve overall cholinergic function in AD patients [16,17]. The ECB device contained genetically modified human retinal pigment epithelial cell line (ARPE-19), which produced and released hmNGF (termed NGC-0295 and NGC-0211). We used ECB-devices containing hmNGF releasing cells (ECB-NGF) in clinical trials in a phase I pilot study [16]. During 6–12 months of implanted ECB-NGF devices and hmNGF-delivery into the basal forebrain of AD patients, we observed increased glucose metabolism, decreased rates of brain atrophy, more stable cognitive function, and improvement of the level of cholinergic markers in the cerebrospinal fluid (CSF) [16,18,19]. This delivery technology showed to be well tolerated with no off-target side effects [17]. In this approach, despite the improvement in several biological activities, we also detected reduced hmNGF release from the ECB devices during the longer time of implantation (1 yr.) as well as the degeneration of NGF-producing cells.

The difficulties in obtaining stable hmNGF release and cell survival in all ECB devices in our clinical trials for AD therapy are likely due to multiple factors [20,21]. Apart from several other factors, induced gliosis and microglial (brain resident macrophages) activation in AD brain causes increased inflammatory responses in both the AD brain and upon device implantation. Therefore, increased inflammatory molecules may have a negative impact on hmNGF-releasing cells. This hypothesis agrees with our previous in-vitro studies, where we demonstrated that inflammatory protein, such as IL-1β, has a negative effect on hmNGF production by ECB cells [22]. Another potential factor may be increased levels of Aβ peptides in the AD brain, which, due to their small size, can penetrate the membrane of the capsules and have an influence on the hmNGF-producing cells inside the capsules, and recently shown to affect NGC0211 cell proliferation in-vitro [23].

In the present study, we aimed to investigate whether activated microglial cells have an impact on hmNGF production from ECB-NGF devices and on the underlying molecular alterations induced in NGC0211 cells.

## 2. Results

### 2.1. The Impact of Aβ on the hmNGF Release from ECB-NGF Devices

Following our previous in-vitro studies [22,23], we continue to investigate the effect of AD-related factors on hmNGF release from ECB-NGF devices and NGC0211 cultures, respectively. Aβ-triggered microglial activation and the resulting inflammation are a hallmark of AD, and the effect on ECB-NGF devices needs to be evaluated. Supplementary data show that in-vitro exposure of HMC3 to Aβ triggers its activation (Figures S1A–C and S2A–E).

**Figure 1:**
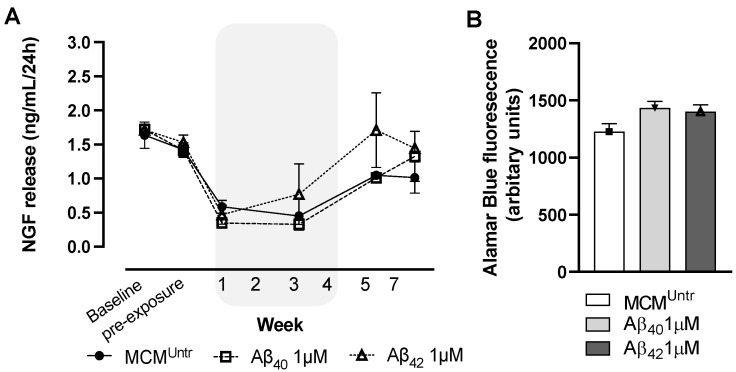
Impairment of hmNGF release from ECB-NGF devices in-vitro. ECB-NGF devices were initially maintained in complete DMEM/F12 medium, and then exposed to various MCMs (MCM^Untr^, MCM_A__β40_, MCM_A__β42_) for consecutive 4 weeks depicted by the shaded background (refer Section 4.6). This was followed by recovery period from week 5 to week 7, where ECB-NGF devices were placed in fresh complete DMEM/F12 media. (**A**) 500 µL media was drawn every week and hmNGF was measured using ELISA. The release of hmNGF at week 1 was found hampered upon exposure to MCM^Untr^, MCM_Aβ40_, and MCM_Aβ42_. Continued incubation with MCM^Untr^, MCM_Aβ40_ and MCM_Aβ42_ maintained low hmNGF release at week 3. Post week 4, the recovery period displayed increased hmNGF release as compared to the treatment period. (**B**) Bar diagram represents the metabolic activity of ECB-NGF devices at week 7, measured by Alamar blue assay (refer to Section 4.9). No significant difference was found between ECBs treated with various MCMs. Data are represented as mean ± SEM (*n* = 2). MCM is the microglia conditioned medium.

Initial estimation revealed a stable hmNGF release from the ECB-NGF devices on baseline and pre-exposure time points, respectively. After one-week incubation, ELISA analyses revealed a considerable reduction in hmNGF release from ECB-NGF devices exposed to MCM^Untr^, MCM_Aβ40_ and MCM_Aβ42_, respectively (Figure 1A). Continued incubation with MCM^Untr^, MCM_Aβ40_ and MCM_Aβ42_ maintained low hmNGF release at week 3.

Post-MCM exposure, at week 5, a recovery was observed from the ECBs exposed to MCM_Aβ40_ and MCM^Untr^, showing increased hmNGF release compared to the levels found during week 3. However, these recovered hmNGF levels at week 5 were still lower than the pre-exposure levels in the MCM_Aβ40_ group. The recovery in the MCM_Aβ42_ group reached comparable levels similar to the pre-exposure levels. Recovered hmNGF release was maintained through weeks 5 and 7, respectively.

At 7 weeks of incubation, Alamar Blue assay was performed to assess whether MCM_Aβ40_, MCM_Aβ42_ and MCM^Untr^ exposure altered the cellular metabolic activity of NGC0211 cells growing within ECB-NGF devices. Upon exposure to MCM_Aβ40_ or MCM_Aβ42_, marginally increased metabolic activity was observed in treated groups compared to MCM^Untr^ (Figure 1B).

### 2.2. The Metabolic Activity of NGC0211 Cells Exposed to MCM^Untr^, MCM_Aβ40_, and MCM_Aβ42_

**Figure 2:**
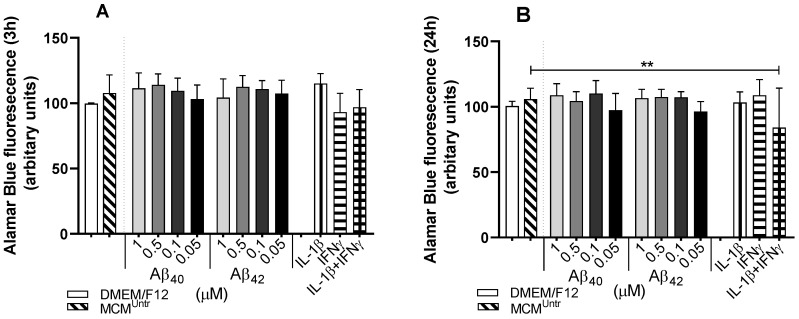
Altered metabolic activity of NGC0211 cells exposed to various MCMs. NGC0211 cells were exposed for 3 or 24 h to various MCMs, and the metabolic activity was analyzed by Alamar Blue assay (refer Section 4.9). Various concentrations of Aβ peptide (1, 0.5, 0.1, 0.05 μM) were utilized to obtain MCMs, and are used to represent in the bar-plots evaluated at (**A**) 3 h and (**B**) 24 h, respectively. Statistical significance was performed using one-way ANOVA analyses with a Tukey’s multiple comparison test to compare experimental control (MCM^Untr^) and other groups. Data are represented as mean ± SEM (*n* = 3). ** *p* < 0.01.

To understand whether the reduction in hmNGF release from ECB-NGF devices was associated with metabolic status in NGC0211 cells, we used Alamar Blue assay. We exposed NGC0211 cells in-vitro to MCMs, (obtained by exposing HMC3 cells to various concentrations of Aβ peptides (1.0, 0.5, 0.1, 0.05 µM), positive controls (IL-1β, IFNγ or IL-1β + IFNγ), untreated (MCM^Untr^)) or the negative control (fresh DMEM/F12) group, respectively (Figure 2).

No significant change in metabolic activity could be seen after 3 h or 24 h exposure to most MCMs or negative control (Figure 2A,B); however, MCM_IFNγ+IL-1β_ led to a significant reduction in metabolic activity after 24 h (*p* < 0.01) as compared to MCM^Untr^ (Figure 2B).

### 2.3. NGC0211 Cells Resists MCM Induced ΔΨ_m_ and Oxidative Stress

**Figure 3:**
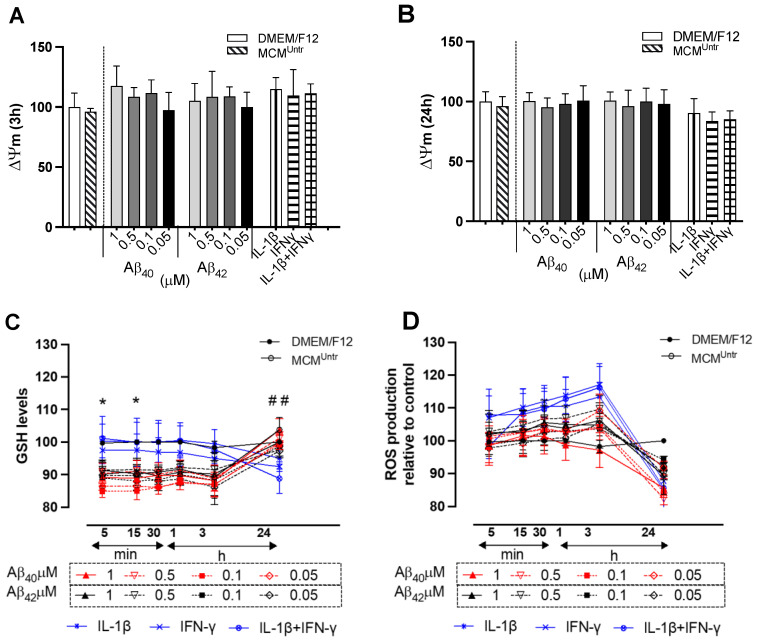
The effect of MCMs on early stress markers. MCMs were obtained by exposing HMC3 cells to various Aβ_40/42_ concentrations (1, 0.5, 0.1, 0.05 μM), and inflammatory cytokines (IL-1β, IFNγ, IL-1β + IFNγ) for 24 h. NGC0211 cells were incubated with different MCMs, and various parameters were measured. (**A**,**B**) represents mitochondrial membrane potential measurements (ΔΨ_m_) at 3 h and 24 h time points, respectively. (**C**) Total glutathione (GSH) content of NGC0211 cells were measured kinetically up to first 1 h of exposure, followed by endpoint measurement at 3 h and 24 h. Data are represented as line-graph displaying various treatment groups along with the experimental control (MCM^Untr^) and negative control (DMEM/F12) group. (**D**) Reactive oxygen species (ROS) content was measured (0–1 h kinetics, 3 h endpoint) and late (24 h endpoint) and displayed as line-graph, respectively. Statistical significance was performed using one-way ANOVA analyses with a Tukey’s multiple comparison test to compare experimental control (MCM^Untr^) and other groups for ΔΨ_m_ analyses. Two-way ANOVA with a Tukey’s multiple comparison test was used to compare experimental control and other groups within each time point for GSH and ROS analyses. Data are represented as mean ± SEM (*n* = 3). * *p* < 0.05, and ^##^ *p* < 0.01. Significance was denoted for specific groups as follows—* MCM_Aβ40_ (0.1 µM), ^##^ IL-1β + IFNγ compared to DMEM/F12.

Collectively, there was no obvious alteration of ΔΨ_m_ in NGC0211 cells exposed to various MCMs at an early (≤3 h) stage of exposure (Figure 3A) but showed a general mild lowering after 24 h in the positive control groups (Figure 3B).

Since mitochondrial activity is directly related to the oxidative status within a cell [24], the antioxidant potential glutathione (GSH content) was examined over time. Exposure to MCM_Aβ40_ (0.1 µM) reduced GSH levels very early, at 5 and 15 min (*p* < 0.05), respectively, but was restored back to the control levels at 24 h (Figure 3C), when compared to the DMEM/F12 group. Although no significant changes were observed in the cellular GSH level after 24 h, MCM_IFNγ+IL-1__β_ exerted a significant reduction (*p* < 0.01) when compared to the DMEM/F12 group. No significant alteration in the GSH content was observed in any of the MCM groups when compared to MCM^Untr^ at the individual respective time-points analyzed.

Mitochondrial activity is also a major source of reactive oxygen species (ROS) [25], and thus we examined the effect of MCMs on the intracellular ROS content in NGC0211 cells. The basal ROS levels showed no difference between DMEM/F12 and MCM^Untr^ groups. There was no significant increase in the intracellular ROS in NGC0211 cells when exposed to other MCM groups at 3 h, and overall ROS levels fell below control levels at 24 h (Figure 3D).

### 2.4. MCMs Fail to Induce Significant Cell Death in NGC0211 Cells

**Figure 4:**
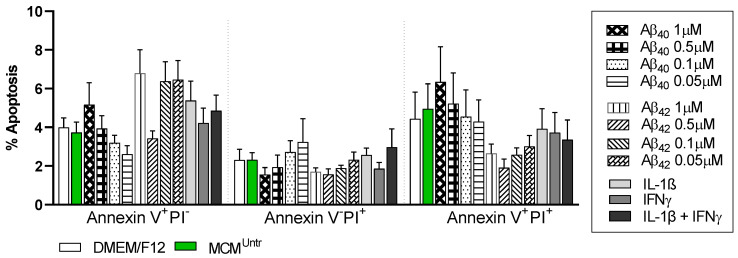
Influence of MCMs on NGC0211 cell death. MCMs were obtained following the exposure of HMC3 to various Aβ_40/42_ concentrations (1, 0.5, 0.1, 0.05 μM), and inflammatory cytokines (IL-1β, IFNγ, IL-1β + IFNγ) for 24 h. NGC0211 cells were exposed to MCMs for 24 h and were stained with FITC-Annexin-V and propidium iodide (PI). Data are represented as mean ± SEM of percent population showing specific staining and categorized as early apoptotic cell death (Annexin V^+^PI^−^), necrotic cells (Annexin V^−^PI^+^), and late apoptotic cells (Annexin V^+^PI^+^). Statistical significance was performed using two-way ANOVA with Tukey’s multiple comparison test to compare control and treated groups (*n* = 3).

To understand whether MCMs affect NGC0211 cell viability, we checked for markers of apoptosis (annexin V) and necrosis (PI). At 24 h, MCM_A__β40_ exerted its toxicity showing higher late apoptotic populations (V^+^PI^+^) (6.7 ± 4%) than MCM_A__β42_ (2.6 ± 3%), and as compared to controls (4.4 ± 3% and 4.9 ± 3% respectively) (Figure 4). Notably, the population of early apoptotic cells (V^+^PI^−^) was higher in NGC0211 cells exposed to MCM_A__β42_ compared to MCM_A__β40_. A dose-dependent effect of MCMs treated with MCM_A__β40_ and MCM_A__β42_ was observed in necrotic cell population (V^−^PI^+^). An overall enhanced effect of Aβ-stimulated MCMs was observed when compared to the positive-control-derived MCMs, although neither of the groups reached statistical significance when compared to MCM^Untr^.

### 2.5. MCMs Significantly Impair Proliferation of NGC0211 Cells

Previously, we showed [23] that exposure to AD-related factors, such as Aβ_40/42_ peptides, or indirect treatment with activated astrocyte-conditioned medium (treated with Aβ_40/42_/IL-1β/TNFα) has an inhibitory effect on the proliferation rate of NGC0211 cells. To examine whether microglia-mediated factors can also display similar effect, NGC0211 cells were exposed to various MCMs or control supernatants for 24 h, labeled with CFSE dye or stained with Ki67 antibody, and performed analyses by flow cytometry and confocal microscopy, respectively.

**Figure 5:**
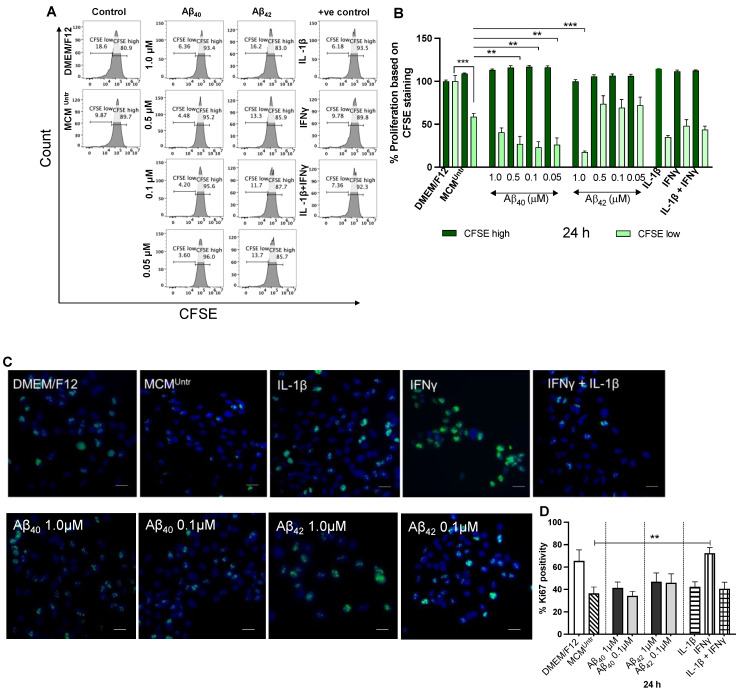
MCMs display anti-proliferative effect on NGC0211 cells. (**A**) NGC0211 cells were stained with 0.5 µM carboxyfluorescein succinimidyl ester (CFSE, 250 µL/well) for 15 min, washed twice with prewarmed complete medium and incubated with appropriate treatment (control media or MCMs) for 24 h followed by data acquisition in a flow cytometer. Representative histogram plot illustrates CFSE fluorescence distribution in NGC0211 cells from individual samples, where *x*-axis presents CFSE fluorescence, and *y*-axis represents cell count. DMEM/F12 group was utilized to fix gating strategy to evaluate dividing cells (CFSE low) group. (**B**) Quantitative bar-plots summarize the flow cytometric data and represents percent change in CFSE fluorescence in comparison to DMEM/F12 group (*n* = 3). CFSE high represents non-dividing cells, whereas CFSE low represents actively dividing cells, respectively. (**C**) NGC0211 cells were exposed to appropriate treatment (control media or MCMs) for 24 h, probed for Ki67 expression, and images were captured using a confocal microscope. Representative images display Ki67 staining (green) and nuclear staining (blue) in NGC0211 cells (scale bar 20 µm). (**D**) Bar diagram presents percent Ki67 positive population in comparison to total cell number in each well and are represented as mean ± SEM (*n* = 3). ** *p* < 0.01, *** *p* < 0.001.

A representative flow-cytometry histogram illustrating CFSE fluorescence in NGC0211 cells after exposure to various MCMs is shown in Figure 5A and the collective data are summarized in Figure 5B. The fluorescence histogram represents two populations of NGC0211 cells based on their CFSE content—‘low CFSE’ representing cells which has divided post-staining and ‘high CFSE’ which has not divided post-staining, respectively. The data show a significant anti-proliferative effect of MCM^Untr^ on NGC0211 cells, as compared to low CFSE containing cells in the DMEM/F12 group, implying the role of factors released from untreated microglia. Furthermore, treatment with MCM_A__β40_ further enhanced this anti-proliferative effect, which now displays a significantly reduced number of cells containing a low CFSE content. Among MCM_A__β42_-treated groups, only the highest concentration of 1 µM showed significant anti-proliferative capacity on NGC0211 cells. Likewise, positive controls (MCM_IFNγ_, MCM_IL-1__β_, and MCM_IFNγ+IL-1__β_) did not show significant effects when compared to the MCM^Untr^ group. Overall, our analysis showed that after 24 h, MCM_A__β40_ had a stronger anti-proliferative effect on NGC0211 cells, higher than MCM_A__β42_ (Figure 5A,B).

In addition, anti-Ki67 staining and confocal microscopy (Figure 5C) showed that after 24 h, a lower number of Ki67 positive cells were found in cultured NGC0211 cells exposed to MCM^Untr^ when compared to the DMEM/F12 control. We did not find significant differences between MCM^Untr^ and other MCM groups, although MCM_A__β40_ (0.1 µM) displayed a stronger anti-proliferative effect than MCM_A__β42_ (Figure 5D). Of note, we observed an inhibitory effect of MCM_IL-1__β_ and MCM_IFNγ+IL-1__β_ but not MCM_IFNγ_ on the proliferation of NGC0211 cells, when compared to the negative control group (Figure 5D).

### 2.6. Cellular Senescence Was Higher in NGC0211 Cells Exposed to MCM_A__β40_

**Figure 6:**
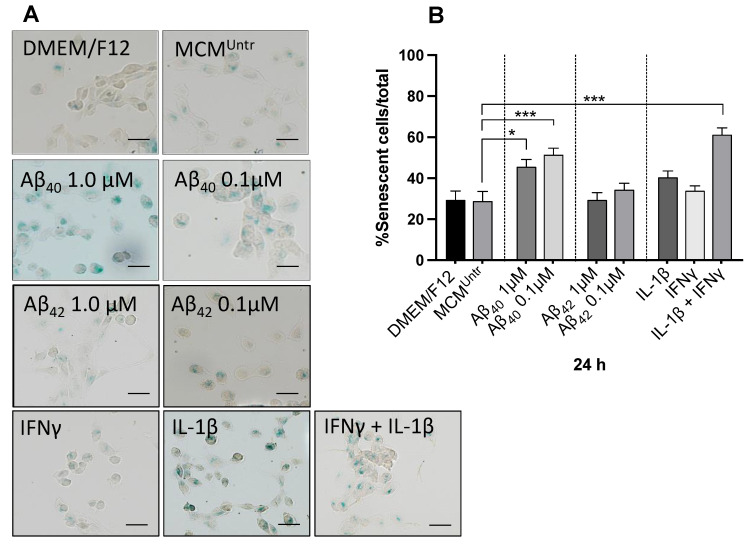
MCM_Aβ40_ induces senescence in NGC0211 cells. NGC0211 cells were exposed to various MCMs for 24 h and stained for senescence associated β-galactosidase (SA-β-gal) activity, seen as blue coloration representing senescent cells. (**A**) Representative images of the SA-β-gal positive NGC0211 cells exposed. (**B**) Quantification of the percentage of senescent cells among total cells within a well and are represented as mean ± SEM (*n* = 3). Statistical significance was performed using one-way ANOVA analyses with a Tukey’s multiple comparison test to compare control (MCM^Untr^) and treated groups. * *p* < 0.05 and *** *p* < 0.001.

To evaluate the effect of various MCMs, we exposed NGC0211 cells for 24 h and stained for the senescence associated β-galactosidase (SA-β-gal) activity, which displays senescent cells. Representative images of the SA-β-gal positive NGC0211 cells exposed to different MCMs and control groups are shown in Figure 6A. Surprisingly, senescent cells in the control groups (DMEM/F12 and MCM^Untr^) were comparable, showing that untreated microglia did not induce NGC0211 cell senescence. The mean percentage of senescent cells per total number of cells showed that the numbers of senescent cells in MCM_Aβ40_ and MCM_IFNγ+IL-1β_ groups were significantly higher than the numbers of such cells in the other groups (Figure 6B). Once again, the impact of MCM_Aβ40_ was found to be stronger than MCM_Aβ42_ on NGC0211 cells.

## 3. Discussion

The physiology of AD is complex, and current disease modifying pharmacological therapies for AD have been largely ineffective. Nonetheless, in recent years, tremendous progress has been accomplished in developing and testing new AD treatments, including several medications that are in late-stage clinical trials. In this study, we utilized in-vitro studies to investigate the effect of AD-related factors on one of the recent therapeutic strategies, encapsulated cell biodelivery (ECB) technology, for mitigating symptoms in AD patients. In the context of using ECB devices as a new therapeutic technology for AD, we previously observed IL-1β as a factor that causes the degeneration of hmNGF-releasing NGC0211 cells, and reduced hmNGF production [22]. As a continuation of previous studies, here we investigated the role of activated microglial cells on affecting the NGC0211 cells. In these experiments, we used the HMC3 cell line, which have the properties of microglial cells [26] and were activated by Aβ peptides (Figures S1 and S2).

We found reduced hmNGF release from ECB devices after exposure to different MCMs. This effect was more apparent and prolonged in devices incubated with MCM_Aβ40_ compared to MCM_Aβ42_. It is important to note that even the MCM^Untr^ supernatant also reduced hmNGF release from ECB-NGF devices, indicating that NGC0211 cells are sensitive to factors released by untreated microglia, as previously shown by other reports [27]. Furthermore, we provided evidence that the activation of the HMC3 cell line with the Aβ40 peptide can contribute to a lower proliferation rate of NGC0211 cells in-vitro. However, we found that none of the conditioned medium from activated human HMC3 cells could alter cell survival or stress parameters in NGC0211 cells.

To date, the functional properties of Aβ_40_ have not been completely elucidated, while most research studies are focused on the pathological function of the Aβ_42_ peptide. In general, it is established that Aβ peptides cause neurotoxicity and may lead to cell death [28]. However, observations from these studies are dependent on peptide concentration (nanomolar to micromolar) used, peptide preparation procedures, incubation time and the structural form of the Aβ peptides, i.e., oligomeric or fibrillar [29]. Importantly, the physiological concentration of the Aβ peptides in brain tissue reaches around 1 µM in AD brain [30]. To this point, we used soluble oligomeric form of Aβ-peptides within the physiological concentration limit for all experimental procedures to mimic clinically relevant concentrations in AD brain. Increased production of Aβ peptides and their probable diffusion across the ECB device membrane due to their small molecular size may be a potential factor involved in the degeneration of encapsulated cells.

One of the significant findings in the current study was the inhibitory effect of MCMs obtained after incubation with Aβ peptides on the proliferation rate of hmNGF-producing cells. Our current study, along with the recently published paper [23], raise new questions about the mechanisms whereby Aβ peptides affect the proliferation of the NGF-producing cells. Do peptides have a direct or indirect effect on the proliferation process? Additional questions remain to be answered whether there are other factors in combination with Aβ peptides that initiate the activation of other functions, such as the up-regulation or down-regulation of key genes involved in the proliferation rate of the cells inside the capsules. For instance, it has been reported that a high Aβ_40_ concentration (25 µM) can cause an inhibitory effect on ARPE-19 viability and proliferation [31]. However, this effect could be due to the toxicity of high concentrations of Aβ peptides, considering that the physiological concentrations of the peptides are relatively low in the brain tissue. Interestingly, another study demonstrated that the apoptotic bodies of ultraviolet A (UVA) irradiated ARPE-19 cells, suppressed the proliferation of ARPE-19 cells in a dose-dependent manner, caused quantitative depressions in the transmembrane potential (ΔΨ_m_) and induced both early and late apoptosis [32]. There is a possibility that increased numbers of apoptotic cells in the capsule can affect the hmNGF-producing cells and their proliferation.

Adding to these observations, the MCM_A__β40_ triggered senescence in the NGC0211 cells. Senescence arrests cell division, and cellular senescence can be initiated by a wide variety of stress-inducing factors. These stress factors include environmental conditions, internal damaging events, abnormal cellular growth, oxidative stress, and autophagy factors [33]. The senescent assay demonstrated that the numbers of senescent positive NGC0211 cells after exposure to MCM_IL-1β_, and MCM_IFNγ+IL-1β_ were increased, compared to MCM^Untr^. Interestingly, MCM_IFNγ_ treatment alone showed lower numbers of NGC0211 senescent cells. Yet, when we used MCM_IFNγ+IL-1β_ (combined IFNγ and IL-1β treatment to HMC3), the number of senescent positive NGC0211 cells were increased. This indicates that IL-1β might influence the senescent process in NGC0211 cells by an unknown mechanism, which needs to be further elucidated. However, it should be noted that in the AD brain, there is mixture of many cytokines, which may have a variety of effects on ECB device cells, which may invite further research.

Due to the heterogeneous nature of senescent cells, different immune cells eliminate different senescent cells [34,35]. In a recent study, the involvement of immune cells on encapsulated cells was described [36]. Utilizing an in-vitro setting, the research group demonstrated that antigens shedding from encapsulated cells can be presented to antigen-presenting cells after diffusing out of the hydrogel, leading to indirect antigen recognition and subsequent T-cell activation. Consequently, the perforin released from activated T cells can penetrate the membrane of the ECB devices and form pores on the target cells, allowing the granzymes (serine proteases released by cytoplasmic granules within cytotoxic T cells) to enter the target cells, which leads to cell death. In our experimental set-up, we observed HMC3 activation (Figure S2A) upon exposure to Aβ-peptide and positive control, which led to complement-C3 release (Figure S2D), although direct contact between microglia and ECB-NGF devices was not tested (this is out of scope of this manuscript and will be addressed in future studies).

Growing evidence suggest that the microglia-mediated inflammatory response have both harmful and/or beneficial functions [37,38]. Considering the increased levels of microglia activation in the AD brain, we tested whether activated microglia could have an impact on the hmNGF-producing NGC0211 cells. Increased concentration of inflammatory mediators is likely dependent on the high infiltration of activated glial cells around the capsules in the AD brain. Continual high concentrations of inflammatory molecules and soluble Aβ peptides can damage the function of hmNGF-producing cells inside the ECB devices, possibly with incremental intensity over time. Moreover, findings related to the impact of the IL-1β cytokine on hmNGF-producing cells from a previous study [22], in conjunction with the effect of IL-1β on astrocytes [23], provide additional evidence of an inhibitory effect of inflammation on the proliferation rate of NGC0211 cells and reduced hmNGF-release, either directly or indirectly. In fact, it was reported that the IL-1 family mediates inflammation and cell death in retinal degeneration [39]. Interestingly, using animal models, an association of IL-1β dysfunction with excessive inflammation in retinal degenerations has been found [40]. Several studies have demonstrated that the inhibition of IL-1β using both small interfering RNA (siRNA) and a neutralizing antibody ameliorated degeneration of retinal pigment epithelial (RPE) cells [41]. Interestingly, we previously showed that the blockage of IL-1β receptor with IL-1Ra antibody in cells treated with IL-1β significantly enhanced the NGF-production [22]. It should be noted that we did not investigate the effect of anti-inflammatory cytokines on hmNGF-producing cells and NGF-release.

In a recent study, the effect of inflammatory cytokines on RPE cells revealed that IFN-γ, tumor necrosis factor alpha (TNFα), and IL-1β decreased the expression of key genes involved in the visual cycle, epithelial morphology, melanogenesis, and phagocytosis in ARPE-19 cells, thus indicating that these proinflammatory cytokines could promote RPE dysfunction [42]. Furthermore, a study by Camelo S et al. observed that APRE-19 dysfunction can be triggered by the inflammatory response, resulting in T lymphocyte infiltration into the brain [43]. Consequently, the activated immune cells engage explicit regulatory mechanisms to eliminate senescent cells [34]. There are also several reports that have highlighted the role of peripheral T lymphocytes in the innate immunity of AD neuroinflammatory processes [44,45]. Since microglia are the resident innate immune cells in the brain tissue, their effect on the ECB-NGF devices when implanted within the brain tissue is relevant to study and is of prime concern.

In this study, Aβ peptides also activated HMC3 cells by increasing inflammatory cytokines, such as IL-6, and a critical component for inflammation, namely component C3 (Figure S2). However, no significant effect of MCMs on the metabolic activity of NGC0211 cells was observed. Activated microglia can produce free radicals, such as ROS [46], which might also disturb hmNGF production from NGC0211 cells during implantation in the AD brain. It is noteworthy that the activated microglia also produce a wide array of neuroprotective factors, including brain-derived neurotrophic factor (BDNF), glial cell-derived neurotrophic factor (GDNF) and NGF [47,48,49] which may help against neuronal injury.

Oxidative stress is a byproduct of mitochondrial respiration and can lead to redox imbalance, neurotoxicity, genomic instability, pro-inflammatory gene transcription and cytokine release (such as IL-1, IL-6 and TNFα) [50]. Mitochondrial function is crucial for the metabolic activity of the cells and its dysfunction has been associated with human aging and neurodegeneration [51]. We here further tested the indirect effect of activated microglia (treated with various AD-related molecules) on cellular stress via biochemical measurements, including the ΔΨ_m_ of NGC0211 cells, we observed a trend of increased mitochondrial hyperpolarization (MHP) induced by different MCMs (Figure 3A,B). Generally, MHP occurs before activation of caspases and, phosphatidylserine (PS) externalization [52]. However, there is a possibility that MHP leads to the dysfunction of oxidative phosphorylation, which disrupts ΔΨ_m_ and damages the integrity of the inner mitochondrial membrane. In addition, the observed MHP in NGC0211 cells might be due to mitochondrial swelling, leading to an increased tetramethyl rhodamine methyl ester (TMRM) signal in MCM-treated NGC0211 cells. We previously reported altered mitochondrial network morphology in hmNGF releasing cells upon stress induction [22,23].

In this study, MHP was observed at 3 h, and surprisingly, the exposure of NGC0211 cells to MCM_Aβ40/42_ did not show any significant change in ROS levels until 3 h but showed significant depletion of GSH levels (Figure 3C,D). On the other hand, exposure to MCM_IFNγ_, MCM_IL-1β_, and MCM_IFNγ+IL-1β_ induced ROS generation without any depletion in GSH levels, indicating a differential effect on NGC0211 cells. After 24 h exposure, the NGC0211 cells exposed to the most MCMs seemed to reverse the imbalance between ROS/GSH, showing decreased ROS production followed by the reinstated GSH-level, except MCM_IFNγ+IL-1__β_, which showed significantly impaired GSH levels and correlated with impaired metabolic activity (Figure 2B and Figure 3C). An early decline in cellular GSH, followed by ROS production, can induce a variety of signals, including apoptotic stimuli. In fact, the analysis of cell death in the current study revealed an increased percentage of apoptotic cell death after 24 h in MCM_Aβ40_ treated NGC0211 cells (Figure 4). Of note, apoptosis analysis demonstrated once more the substantial impact of Aβ_40_ on NGC0211 cells as compared to Aβ_42_ after 24 h exposure [53].

### Limitations of the Study

This study was planned to evaluate the individual contribution of Aβ peptides and inflammatory cytokines in activating microglial cells, and the eventual effect of factors released from microglia on hmNGF-releasing cells in-vitro or those growing within ECB-NGF devices. To achieve these goals, we used an in-vitro experimental set-up which can be a limitation since the physiological interaction of microglia and its activator may differ from in-vivo situation based on the duration of exposure and the different combination of activators involved. Another limitation of using the in-vitro set-up is that it is not equivalent to the conditions within the in-vivo AD brain, thereby possibly altering the activation level of microglial cells. Moreover, we used a human cell line of microglia, whose activity may differ from the primary microglia but again, this was done due to the limited availability of primary microglia cells. Due to the presence of diverse microglial populations within different brain regions, the microglial response toward implanted ECB-devices in-vivo may vary, which can be difficult to address under one in-vitro set-up. Apart from these limitations, we used appropriate controls within our experimental set-up to generate the validated results, signifying the effect of factors released from the microglia on hmNGF-releasing NGC0211 cells.

## 4. Materials and Methods

### 4.1. Preparation of Plasmid

Preparation of the plasmid was described elsewhere in detail [54]. Briefly, HEK293 genomic DNA was PCR amplified and cloned into a pcDNA3.1(+) vector (Invitrogen, Waltham, MA, USA). The resulting vector was modified to insert cytomegalovirus sequences for promoter/chimeric intron from pCI-neo (Promega, Madison, WI, USA) and early enhancer element/chicken beta-actin (CA) promoter sequence from pCAIB (kindly supplied by Ernest Arenas, Karolinska Institute, Solna, Sweden), respectively. Neomycin resistance and hmNGF sequences were then excised from the previous vector and inserted into a modified pT2BH vector (Addgene, Watertown, MA, USA) containing the sleeping Beauty (SB) substrate elements and denoted as pT2.CAn.hNGF.

### 4.2. Generation of hmNGF Expressing NGC0211 Cells and Subsequent Cell Maintenance

Human retinal pigment epithelial cell line, ARPE-19 (CRL-2302, ATCC; Manassas, VA, USA), was cultured under standard conditions of 37 °C and 5% CO_2_ using DMEM/F12 media containing GlutaMAX (Invitrogen, Waltham, MA, USA) and 10% heat inactivated fetal bovine serum (FBS) (Cat no. 0010, Hyclone, Logan, UT, USA), hereafter termed complete DMEM/F12. Cells were cultured continuously and passaged using TrypLE (Life Technologies, Carlsbad, CA, USA), when they reach 90% confluency. For transfection, ARPE-19 cells were co-incubated with pT2.CAn.hNGF along with pCMV-SB-100X (expressing SB transposase without Neomycin cassettes) (Addgene, Watertown, MA, USA) using FuGENE (Roche, Basel, Switzerland), according to the manufacturer’s protocol. G418 (Sigma-Aldrich, St. Louis, MO, USA) was used to select dually transfected single cells, which were then expanded clonally. One of the clones, termed NGC0211, which was found to express a high amount of hmNGF and was previously used by our group in a phase 1b clinical trial in AD patients [55], was selected for the present study. NGC0211 cells to be used for in-vitro experimentation were maintained in culture using the same conditions as described above for the ARPE-19 cultures. NGC0211 cells to be used for the preparation of ECB-NGF devices were maintained in Human Endothelial-serum free medium (HE-SFM, Invitrogen, Waltham, MA, USA) until used.

### 4.3. Preparation of ECB-NGF Devices

Semi-permeable (cut-off of 280 kDa) polysulfone hollow fiber membranes (Gloriana Therapeutics, Warren, RI, USA) were utilized to prepare 7 mm long ECB devices with a diameter of 0.7 mm. The internal space of the devices was threaded with a polyester terephthalate (PET) yarn matrix (Swicofil, Switzerland) to provide an ample surface area for cell growth. Each device was then filled using a semiautomatic custom-made cell injector system (Kineteks, Warwick, RI, USA) with approximately 60,000 NGC0211 cells in a total volume of 6 µL in HE-SFM medium. The open end of the filled devices was then sealed using a photopolymerized acrylic adhesive (Dymax, Torrington, CT, USA), and maintained in 1 mL HE-SFM medium until used for experimentation. Two weeks prior to experimentation, ECB-NGF devices were transferred to DMEM/F12 media (containing 5% FBS) and maintained at 37 °C and 5% CO_2_.

### 4.4. Preparation of Stimulants

In this study, we used AD-associated molecules to induce microglial activation. We used oligomeric amyloid beta (Aβ) peptides (Aβ_40_ and Aβ_42_) and inflammatory cytokines-interferon-γ (IFNγ) and interleukin-1β (IL-1β), respectively.

Briefly, soluble oligomeric Aβ_40_ and Aβ_42_ peptides (rPeptides, Lelystad, The Netherlands) were dissolved in dimethyl sulfoxide (DMSO, Sigma-Aldrich, Sweden), vortexed vigorously, sonicated at 40 Hz (Branson 2510 bath Sonicator, Sigma-Aldrich, Sweden) for 10 min and aliquoted and stored at −20 °C until used. This method of Aβ solubilization has been previously reported to prevent the formation of fibrillar forms of Aβ under cell culture conditions [56]. Although peptides expressed in *E. coli* do not contain post-translational modifications found on mammalian proteins, they do provide a reliable source of peptide of consistent quality, especially important when considering the various structures of Aβ peptides reported in in-vitro, pre-clinical and clinical studies [57].

Prior to microglia stimulation, fresh Aβ aliquots were directly diluted with FBS-free DMEM/F12 medium to working concentrations (1.0, 0.5, 0.1, 0.05 µM) maintaining <0.1% DMSO final concentration. Similarly, IFNγ (R&D Systems Inc., Minneapolis, MN, USA), IL-1β (R&D Systems Inc., USA) or IFNγ + IL-1β combination were directly diluted in FBS-free DMEM/F12 medium to their final working concentrations of 10 ng/mL each, respectively [26].

### 4.5. Microglia Cell Culture and Preparation of Microglial Conditioned Media (MCM)

Human microglia cell line (HMC3) (CRL-3304, ATCC; Manassas, VA, USA) was grown in complete DMEM/F12 media in T-75 flasks (Corning, New York, NY, USA) and maintained under 5% CO_2_ and 37 °C. The cells were passaged using TrypLE two to three times per week until used for experiments (up to passage 15).

For the microglial-conditioned medium (MCM) preparation, HMC3 cells were passaged, and 7 × 10^4^ cells/well/500 µL were plated in 24-well plates (Corning, New York, NY, USA) for 24 h in complete DMEM/F12 medium. The medium from each well was then discarded, and specific stimulants (Aβ_40_, Aβ_42_, IFNγ, IL-1β, IFNγ + IL-1β) were introduced to the respective wells in 500 µL of FBS-free DMEM/F12 media. Stimulation was performed for 24 h followed by the collection of the conditioned media, centrifuged at 3000× rpm for 10 min to remove floating microglial cells, double diluted with pre-warmed complete DMEM/F12 media (5% FBS final concentration) and used directly for the stimulation of NGC0211 cells or ECB-NGF devices, respectively. The respective condition media are denoted as follows: MCM_A__β40_, MCM_A__β42_, MCM_IFNγ_, MCM_IL-1__β_, and MCM_IFNγ+IL-1__β_, respectively. HMC3 cells treated with DMSO (0.1% final concentration) provided control for factors released by unstimulated microglia and denoted as MCM^Untr^. It is well known that, even under physiological conditions, microglia can release different cytokines [27]. MCM^Untr^ itself serves as an ‘experimental control’ for additional factors, which might be released upon stimulation by activated microglia. Likewise, to control for MCM^Untr^, we employed a negative control, where NGC0211 cells were kept in fresh DMEM/F12 medium containing 5% FBS-denoted ‘DMEM/F12’ group. The negative control provides a reference group for the effect of factors that might be released from untreated microglia (MCM^Untr^).

### 4.6. Treatment of NGC0211 Cells and/or ECB-NGF Devices with MCMs

In this study, all experiments were performed by exposing NGC0211 cells and/or ECB-NGF devices to various MCMs. NGC0211 cells and ECB-NGF devices were then evaluated for various experimental endpoints.

NGC0211 cells were grown by using DMEM/F12 media containing 5% FBS for 24 h in either 96-well plates in a final volume of 100 µL (biochemical assays), 24-well plates in a final volume of 500 µL (apoptosis assay, flow cytometry) or 16-well chamber slides in a final volume of 100 µL (immunohistochemistry, and senescence assay), using a different cell number according to the experimental set-up, respectively (described in detail under each section below). The medium was then discarded, and NGC0211 cells were treated (with control media—fresh DMEM/F12 media containing 5% FBS; or double-diluted MCMs containing 5% FBS) for 24 h, and the resulting culture supernatant was subsequently processed as per experimental need, which is described in the subsequent sections.

ECB-NGF devices were transferred to DMEM/F12 medium (containing 5% FBS) 2-weeks prior to experiment initiation. Devices were maintained every week in 24-well plates using 1 mL media, after which old media was replaced with 1 mL fresh medium. After 4 h incubation with fresh media, 500 µL supernatant was withdrawn and saved at −80 °C for future hmNGF analysis (baseline samples), and additional 500 µL fresh medium was added to make up the final volume to 1 mL. Similarly, after 1 week incubation, the medium was again replaced with 1 mL fresh medium, and after 4 h incubation, 500 µL supernatant was withdrawn and saved at −80 °C (pre-exposure samples). Baseline and pre-exposure samples indicate the healthy status of the devices prior to MCM exposure. At this point, the remaining medium was discarded from each well, and 1 mL of respective MCMs (MCM^Untr^, treated with 1 µM Aβ_40_ or Aβ_42_, respectively) were added as treatment for 1 week. For every passing week, 500 µL supernatant was saved as mentioned earlier and the MCM treatment was continued until week 4. At this point, after collection of 500 µL supernatant, ECB-NGF devices were put on fresh DMEM/F12 medium (containing 5% FBS) to check their recovery from the MCM treatment. ECB-NGF devices were maintained for an additional 3 weeks in fresh medium, and 500 µL supernatant was collected every week to estimate the hmNGF release.

### 4.7. hmNGF Release Estimation

Culture supernatant from ECB-NGF devices following treatment with MCMs were utilized to measure the total hmNGF content using ELISA kit (R&D Systems; Cat No. DY256), with minor modifications as previously described [23]. Briefly, 384-well plates (MaxiSorp, Thermofisher, Waltham, MA, USA) were coated with 50 μL/well capture antibody (2.0 μg/mL in carbonate buffer, pH 9.8) overnight at 4 °C, washed once with 100 μL/well tris-buffered saline (TBS), and blocked for 1 h with 5% bovine serum albumin (BSA; 100 μL/well, prepared in carbonate buffer). Wells were then washed 3× with 100 μL/well TBS-Tween20^0.05%^ (TBS-T) and incubated overnight with 50 μL of respective samples or standards (S1–S10 in a two-fold serial dilution; S1 = 2 ng/mL) at 4 °C. Plates were then washed 3× with 100 μL/well TBS-T and incubated with 50 μL/well NGF detection antibody for 3 h at room temperature (RT). Plates were again washed 3× with 100 μL/well TBS-T and incubated 1 h at RT with 50 μL/well streptavidin-alkaline phosphatase (Streptavidin-AP, Roche Diagnostics; 1:10,000 dilution). Plates were then washed 2× with 100 μL/well TBS-T followed by 1× wash with diethanolamine buffer (1 M, pH 9.8). Then, 50 μL/well of substrate for alkaline phosphatase was added to each well, and the absorbance was kinetically monitored for 1 h with 5 min interval at 540 nm in a spectrophotometer (Safire II, Tecan, Männedorf, Switzerland). The amount of hmNGF was calculated from a standard curve.

### 4.8. Biochemical Assays

#### 4.8.1. Reactive Oxygen Species (ROS) and Total Glutathione (GSH) Measurement

Intracellular reactive oxygen species (ROS) generation and glutathione (GSH) extinction are early markers of stress, and play a major role in initiating the oxidative damage in cells. We used 20 µM dichlorodihydrofluorescein diacetate (DCFH-DA; excitation/emission—485/520 nm) to measure ROS and 50 µM monochlorobimane (mBCL; 394/490 nm) to measure GSH (Invitrogen, Waltham, MA, USA), respectively. For early time point measurements, NGC0211 cells (1 × 10^4^ cells/well/100 µL) were cultured for 24 h in clear bottom black 96-well plates (Corning, New York, NY, USA) and pre-incubated with either DCFH-DA or mBCL for 20 min. Cells were then treated with control media or MCMs and fluorescence kinetic readings were immediately started in a spectrophotometer (Safire II Plate reader, Tecan; 5 nm bandpass; bottom read) for the first one hour. The plates were then returned to an incubator maintained at 5% CO_2_ and 37 °C and read after the completion of 3 h incubation. To study the effect after 24 h exposure, separate plates containing NGC0211 cells (1 × 10^4^ cells/well/100 µL) were adequately treated, DCFH-DA or mBCL was added 30 min prior to completion of treatment incubation time, and endpoint readings were obtained using the spectrophotometer, as mentioned above.

#### 4.8.2. Mitochondrial Membrane Potential (ΔΨ_m_)

Mitochondrial membrane potential (ΔΨ_m_) serves as a sensitive measure of mitochondrial activity and physiological status. NGC0211 cells (1 × 10^4^ cells/well/100 µL) were plated onto clear bottom 96-well black plates (Corning, New York, NY, USA) and allowed to grow for 24 h. Cells were then treated with the control medium or MCMs for 3 or 24 h, respectively. At 30 min prior to treatment completion, 0.2 µM tetramethyl rhodamine methyl ester (TMRM, final concentration) (Invitrogen, Waltham, MA, USA) was added. Cells were then washed twice with 100 µL phosphate-buffered saline (PBS) (Invitrogen, Waltham, MA, USA), and the fluorescence at 548/574 nm was read using a spectrophotometer (Safire II Plate reader, Tecan, Männedorf, Switzerland; 5 nm bandpass; bottom read).

### 4.9. Metabolic Activity Assay

Alamar Blue (Invitrogen, Waltham, MA, USA) was used to evaluate the overall metabolic status of ECB-NGF devices or NGC0211 cells. Briefly, NGC-0211 cells (1 × 10^4^ cells/well/100 µL) were seeded in clear bottom 96-well black plates (Corning, New York, NY, USA) for 24 h and exposed to control media or MCMs for 3 and 24 h, respectively. In the meantime, 10 µL of 10× Alamar blue was added to every well during the last 1 h of treatment incubation. Fluorescence was then read in a spectrophotometer (Safire II Plate reader, Tecan, Männedorf, Switzerland; 5 nm bandpass; top read) at 560/590 nm. Similarly, post 7 weeks of experimentation with ECB-NGF devices, 50 µL of 10× Alamar blue was added in 500 µL DMEM/F12 medium containing 5% FBS and incubated for 1 h. From each well, 100 µL was drawn in triplicate, plated in black bottom 96-well plate (Corning, New York, NY, USA) and the fluorescence was read as mentioned above.

### 4.10. Flow Cytometric Estimation for Cell Death

To check whether MCMs could induce cell death in the NGC0211 cells, we used Vybrant Apoptosis Assay Kit (V13241, Invitrogen, Molecular Probes, Waltham, MA, USA), according to the manufacturer’s recommendation. Briefly, NGC0211 cells (7 × 10^4^ cells/well/500 µL) were seeded for 24 h in 24-well plates, followed by exposure to control media or MCMs for another 24 h, respectively. Post-exposure, culture supernatant was collected and centrifuged at (3000× *g* rpm, 4 °C, 10 min) to obtain the floating cells, which are usually dead cells. Simultaneously, the adherent cells were detached using TrypLE and collected following centrifugation at 2000× *g* rpm for 10 min at 4 °C. Adherent cell pellets were resuspended in 1× annexin binding buffer and pooled with cell pellets obtained from the floating cells in previous steps, thereby collectively representing the total cell content within a sample. Collected cells were then stained with Annexin-V-FITC and propidium Iodide (PI), according to the manufacturer’s instructions and acquired in flow cytometer (Accuri C6, BD Biosciences, San Jose, CA, USA). Depending on the binding patterns of the probes, cells were designated as follows: viable cells (Annexin V^−^ PI^−^), early apoptotic cells (Annexin V^+^PI^−^), late apoptotic cells (Annexin V^+^PI^+^) and necrotic cells (Annexin V^−^PI^+^).

### 4.11. Estimation of Cell Proliferation

#### 4.11.1. Flow Cytometric Evaluation of CFSE Staining

Carboxyfluorescein succinimidyl ester (CFSE; V12883 ThermoFisher, Waltham, MA, USA) is cell permeable and used to detect cell proliferation, according to manufacturer’s recommendation. Fluorescence decreases after every cell division, as only around half of the labeled proteins are given to the subsequent daughter cell [58]. To evaluate the proliferation kinetics of NGC0211 cells, they were plated (7 × 10^4^ cells/well/500 µL) for 24 h in 24-well plates, washed with prewarmed PBS twice and incubated with 0.5 µM CFSE (250 µL/well in PBS) for 15 min at 5% CO_2_ and 37 °C. Cells were then washed twice in prewarmed complete medium and incubated with appropriate treatment (control media or MCMs) for 24 h at 5% CO_2_ and 37 °C. Cells were then trypsinized and resuspended in PBS + 0.5% BSA buffer for acquisition in a flow cytometer (Accuri C6, BD Biosciences, San Jose, CA, USA) using the FITC channel. Unstained cells were used as controls to differentiate between CFSE signal and background noise.

#### 4.11.2. Immunocytochemistry for Ki67 Expression

To study the proliferative status of NGC0211 cells, we analyzed the expression of Ki67 protein which is well known to be associated with cell proliferation [59]. Briefly, NGC0211 cells (0.5 × 10^4^ cells/well/100 µL) were plated in 16-well chamber slide (Lab-Tek, ThermoFisher Scientific, Waltham, MA, USA) and subsequently exposed to control or respective MCM medium for 24 h at 5% CO_2_ and 37 °C. Cells were then washed 3× using prewarmed PBS, fixed in 10% formalin (Sigma-Aldrich, St. Louis, MO, USA) for 5 min at RT and permeabilized for 15 min using 0.2% Triton X-100 (Sigma-Aldrich, St. Louis, MO, USA). After 3× wash with PBS+ 0.05% Tween 20 (PBS-T, 5 min each), cells were blocked for 30 min using 1% BSA and stained overnight at 4 °C with 100 µL of mouse anti-human Ki67 antibody (clone MIB-1, DAKO, Glostrup, Denmark; 1:100 dilution in PBS-T + 1% BSA staining buffer). Cells were then washed 3× with PBS-T, blocked for 15 min with 3% goat serum prepared in staining buffer and probed with FITC-conjugated goat anti-mouse secondary antibody (Invitrogen, Waltham, MA, USA) for 2 h at RT. Cells were finally washed 3× with PBS-T followed by air drying and mounting with DAPI containing mounting medium (VectaShield, Vector Laboratories Inc., Oxford shire, UK). Images of the stained cells were captured (LSM 510 META; Zeiss, Oberkochen, Germany); multiple images from sister wells of each sample were counted (at least 500 cells/well) (Adobe Photoshop, San Jose, CA, USA) for Ki67 expression and represented as percentage of Ki67 expression cells against total population.

### 4.12. Senescence Assay

Cell senescence has been widely known to represent alteration in cellular activity in retinal pigment epithelial cells [60,61] and can be measured sensitively by probing for the senescence associated lysosomal marker beta-galactosidase (SA-β-gal). We measured SA-β-gal in control and MCM treated NGC0211 cells using a commercially available kit (KAA002, Chemicon, Sigma-Aldrich, St. Louis, MO, USA), according to the manufacturer’s recommendation. Briefly, NGC0211 cells (0.5 × 10^4^ cells/well/100 µL) were plated in 16-well chamber slide and subsequently exposed to control or respective MCM medium for 24 h at 5% CO_2_ and 37 °C. Post-treatment cells were washed once in PBS and then fixed with 1× fixing solution for 15 min at RT. Cells were then washed twice with PBS and incubated with freshly prepared 1× SA-β-gal detection solution for 24 h at 37 °C in a moist chamber. Cells were then again washed twice with PBS, air dried and mounted (Fluoromount-G, ThermoFisher, Waltham, MA, USA). Images of respective wells were acquired in a light microscope (Nikon Eclipse E800 microscope; Nikon Europe B.V., Amstelveen, The Netherlands), and the stained cells were counted (Adobe Photoshop, San Jose, CA, USA) and represented as percentage of stained cells against the total number of cells in the respective image.

### 4.13. Statistical Analysis

Data are presented as mean ± SEM for all the experiments. Statistical analyses were performed using Prism8 software (Version 8.4.3., GraphPad, San Diego, CA, USA). Data were analyzed by either one-way ANOVA or two-way ANOVA followed by Tukey’s multiple comparison test. A value of *p* = 0.05 was considered as significant.

## 5. Conclusions

Our study provides basic understanding of the challenges in using encapsulated cells as therapeutic tools to deliver drugs in the AD brain tissue. We show that microglia cells (whether activated or not) can reversibly impair hmNGF release from ECB-NGF devices, which may be related to altered cellular function and senescence induction among NGC0211 cells. These observations call for the careful understanding of the factors in the diseased brain, which may alter the therapeutic efficacy of cell-based drug delivery. Additional modification in NGC0211 cells to render them resilient toward microglial factors may enhance hmNGF release over time and aid in future clinical applications.

## Data Availability

The datasets used and/or analyzed during the current study are available from the corresponding author on reasonable request.

## Supplementary Materials

The supporting information can be downloaded at: https://www.mdpi.com/article/10.3390/ijms23169011/s1.

## Abbreviations

AD: Alzheimer’s Disease; Aβ: amyloid beta peptide; AMD: age-related macular degeneration; AnnexinV/PI: annexin-V/propidium iodide; BFCN: basal forebrain cholinergic neurons; CSF: cerebrospinal fluid; CFSE: carboxyfluorescein succinimidyl ester; DCFH-DA: 2′-7′dichlorofluorescin diacetate; DMSO: dimethyl sulfoxide; ECB: encapsulated cell biodelivery; ECB-NGF: encapsulated cell biodelivery-NGF device; ELISA: enzyme-linked immunosorbent assay; FBS: fetal bovine serum; GSH: glutathione; hmNGF: human mature NGF; HMC3: human microglial clone 3 cell line; IL-1β: interleukin-1beta; IFNγ: interferon gamma; MCM: microglia conditioned media; mNGF: mature NGF; MHP: mitochondrial hyperpolarization; mBCL: monochloromobimane; NGF: nerve growth factor; proNGF: precursor form of NGF; RPE: retinal pigment epithelial; ROS: reactive oxygen species; SA-β-gal: senescence associated beta-galactosidase; TMRM: tetramethylrhodamine, methyl ester; TNFα: tumor necrosis factor alpha; TrkA: tropomyosin receptor kinase A; ΔΨ_m_: mitochondrial membrane potential.
