# Supplementary material for: Microglia Impairs Proliferation and Induces Senescence In-Vitro in NGF Releasing Cells Used in Encapsulated Cell Biodelivery for Alzheimer’s Disease Therapy
Source: Int J Mol Sci. 2022 Aug 12;23(16):9011. doi: 10.3390/ijms23169011 (PMC9409429; doi:10.3390/ijms23169011)
Supplement: Supplementary file 1 [file ijms-23-09011-s001.zip › ijms-1836273-supplementary.pdf]

Supplementary

Materials and methods

Measurement of interleukin 6 (IL-6) by ELISA

Released IL-6 in the medium from cultured HMC3 cells was analyzed using human IL-6 ELISA Kit High Sensitivity (ab46042, Abcam, Cambridge, UK), according to the manufacturer's protocol with minor modification. Briefly, the assay was performed by adding 100 µL of standard, control, or sample to the plate. Subsequently 50 µL of 1× biotinylated anti-IL6 antibody was added to all wells. The plate was covered with a plate sealer and incubated at room temperature (RT) for 3 h. Later, each well was aspirated, washed with 300 µL 1× wash buffer, and the process was repeated 3 times. The samples wells were then incubated with 100 µL of streptavidin conjugated alkaline phosphatase to each well and incubated 2 h at RT. Sample wells were then washed 3 times with 1× wash buffer, and a final wash with DEA buffer (1M Diethanolamine buffer, pH 9.8). The alkaline phosphatase substrate (p-Nitrophenyl-Na_2-$6H_2O$, 1 mg/mL in DEA buffer) was then added 100 µL/well and colorimetric data was acquired kinetically in a pate reader (Safire II, Tecan) every 5 min for 1 h at 540 nm. Data were analyzed from standard curve.

Complement 3 (C3) measurement by ELISA

The complement-C3 human ELISA Kit (ab108823, Abcam, Cambridge, UK) was used to detect C3 in the MCM (microglia condition medium), according to manufacturer's protocol with minor modification. Briefly, the conditioned media samples from cultured HCM3 cell were centrifuged for 5 min at 5000 rpm at 4°C and added to the precoated wells (96-well). After an overnight incubation at 4°C, the plate was washed 3× with washing buffer followed by incubation with biotinylated complement-C3 antibody and incubated at RT for 2 h. Subsequently, the plate was washed, and the streptavidin conjugated alkaline phosphatase was added and incubated for 2 h. later the plate was washed twice with washing buffer followed by one wash using DEA buffer (pH= 9.8). Finally, the substrate 4-nitrophenyl phosphate disodium salt hexahydrate (1 mg/mL) was added, and the absorbance (405 nm) was measured kinetically every 5 min up to 1 h in a pate reader (Safire II, Tecan) at 540 nm. Data were analyzed from standard curve.

Result and Discussion

1. *Activation of human microglia cell line after Aβ exposure.*

It has been previously reported that synthesis, and release of pro-inflammatory cytokines such as IL-6, may be important in the pathogenesis of Alzheimer's disease (AD) {Guzman-Martinez, 2019 #57}. Further, once activated, microglia are known to release the proinflammatory cytokine interleukin-1β (IL-1β) which is found to be highly expressed in AD. Excessive production of pro-inflammatory cytokines such as IL-6 or IL-1β from activated microglia may affect hmNGF production and release from NGC0211 cells which has been found in activated astrocytes {23}.

We performed tests to confirm whether the HMC3 activation profile was not dependent on other factors in the cultured cells, other than the intended stimulations of AD associated factors. Although ATCC recommends minimal essential medium (MEM) for sub-culturing HMC3 cells, various studies have cultured HMC3 in different media compositions including DMEM/F12 and MEM {26}. To check which media is optimal in culturing HMC3, we compared the release of IL-6 in cells cultured in DMEM/F12 and MEM. Analysis showed that there was no difference of IL-6 release between DMEM and MEM medium (Fig. S1A), thus we chose DMEM/F12 as favorable medium for our study. This is because NGC0211 cells were also grown in DMEM/F12, and this minimizes the effect of variable component of media during NGC0211 cell exposure to HMC3 supernatant. The level of IL-6 was also low in DMEM/12 cell culture medium without serum (Fig. S1B), which was independent from alterations in metabolic activity of the cells(Fig. S1C). Thus, we chose to stimulate HMC3 cells in DMEM/F12 FBS-free media to obtain microglia conditioned medium (MCM) for subsequent experiment.

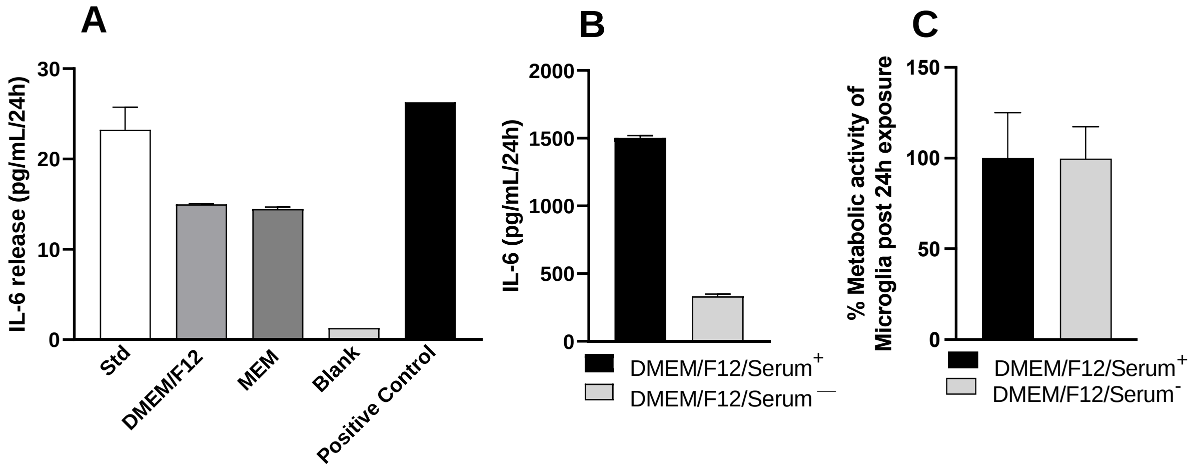

Figure S1. Optimization of HMC3 culture conditions. To verify that HMC3 activation was not dependent on other factors in the culturing condition, several control experiments were performed. (**A**) Basal activation status of HMC3 was compared when cultured in different FBS-free medium – DMEM/F12 or MEM for 24 h, followed by media collection and IL-6 estimation by ELISA. Samples were diluted (50 ×) with FBS-free culture medium to be within the detection range of the kit. (**B**) To check the effect of serum in HMC3 activation, we culture HMC3 in FBS-containing and FBS-free DMEM/F12, followed by media collection and IL-6 estimation by ELISA. IL-6 levels calculated against the standard curve was multiplied by the dilution factor and displayed. (**C**) To make sure that difference in IL-6 release observed in (B) was not due to alteration in cellular function, metabolic activity was measured using Alamar blue assay in HMC3 cells. Data is represented as mean ± S.E (*n* = 2)

2.$A\beta_{40/42}$ *peptides increased IL-6 and complement-C3 release in HMC3*

Having established the culturing method for HMC3, we investigated the ability of AD associated molecules like Aβ-peptides and inflammatory cytokines to activate HMC3 cells. As markers for HMC3 activation, IL-6 release (Fig. S2A-S2C) and complement C3 release (Fig. S2D-E) were evaluated. Examination of IL-6 and complement C3 release from HMC3 cells exposed to Aβ-peptides ($A\beta_{40}$ and $A\beta_{42}$;1.0, 0.5, 0.1, 0.05 μM) or cytokines (IFNγ, IL-1β or IL-1β + IFNγ), revealed an increased level of IL-6 or complement C3 release compared to untreated cells, indicating activated state of HMC3. Moreover, a dose-dependent response was seen after exposure to varied concentrations of Aβ-peptides (Fig.S2A). Notably, activation of HMC3 cells assessed by C3 release was dose dependent for $A\beta_{40}$ but not for $A\beta_{42}$ peptide (Fig. S2D). Further confirmation of the activation ability was seen by the increased C3 levels after cell exposure to positive controls (Fig. S2E).

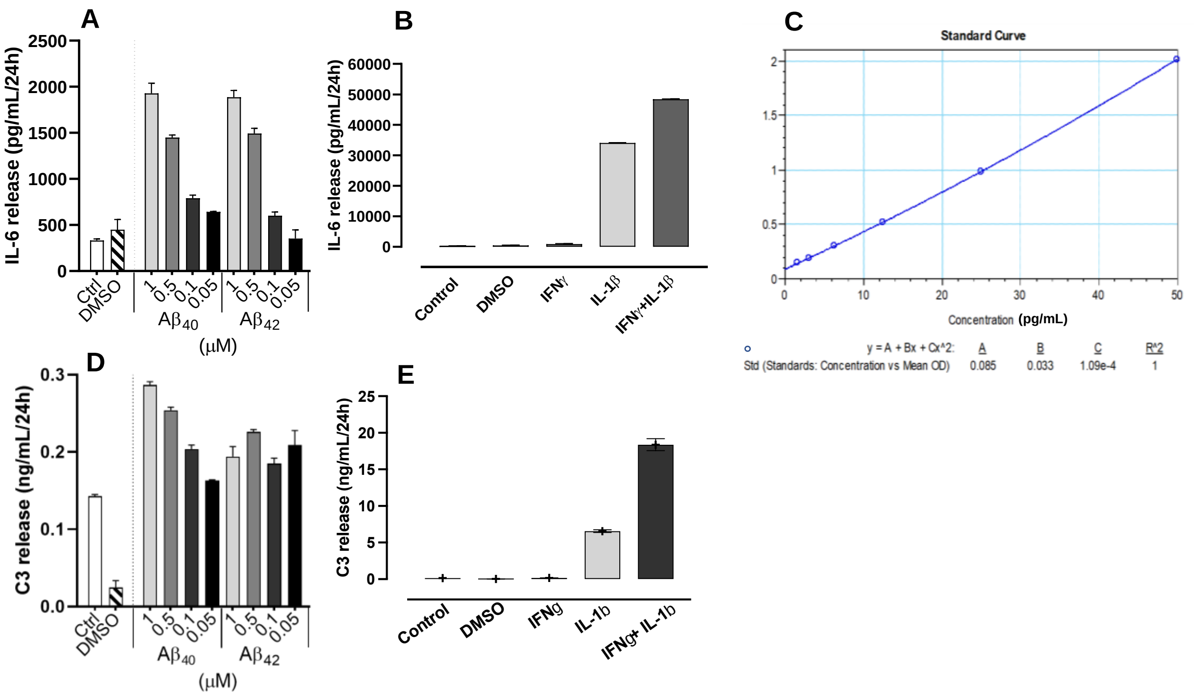

Figure S2. Assessment of HMC3 activation post-stimulation. To ascertain whether AD associated factors like Aβ-peptides (Aβ_{40} and Aβ_{42}) or inflammatory cytokines (IFNγ, IL-1β, or IL-1β + IFNγ) can activate HMC3 cells, stimulation was done in FBS-free medium for 24 h. HMC3 were stimulated with various concentrations of Aβ-peptides (1.0, 0.5, 0.1, 0.05 μM), cytokines (0.01 μg/mL final concentration), or DMSO (< 0.1% final concentration) for 24 h or left untreated (control). Supernatant was recovered post 24 h and activation markers like IL-6 and complement C3 release were checked using ELISA. (A) IL-6 release from HMC3 cells exposed to DMSO or Aβ-peptides; (B) IL-6 release from HNC3 exposed to DMSO or inflammatory cytokines; (C) Standard curve generated by plotting known amount of purified IL-6; (D) Complement C3 release from HMC3 cells exposed to DMSO or Aβ-peptides; (E) Complement C3 release from HNC3 exposed to DMSO or inflammatory cytokines. Data is represented as mean ± S.E (n = 2)
